# CAM and Pediatric Oncology: Where Are All the Best Cases?

**DOI:** 10.1155/2013/632351

**Published:** 2013-08-25

**Authors:** Denise Adams, Courtney Spelliscy, Leka Sivakumar, Paul Grundy, Anne Leis, Susan Sencer, Sunita Vohra

**Affiliations:** ^1^CARE Program, Department of Pediatrics, Faculty of Medicine & Dentistry, University of Alberta, Edmonton General Hospital, 8B19-11111 Jasper Avenue, Edmonton, AB, Canada T5K 0L4; ^2^Faculty of Medicine & Dentistry, University of Alberta, Edmonton, AB, Canada T6G 2R3; ^3^Department of Pediatrics, Faculty of Medicine & Dentistry, University of Alberta, Edmonton, AB, Canada T6G 2R3; ^4^College of Medicine, University of Saskatchewan, Saskatoon, SK, Canada S7N 5E5; ^5^Hematology/Oncology, Children's Hospitals and Clinics of Minnesota, 2525 Chicago Avenue, Minneapolis, MN 55404, USA; ^6^CARE Program, Department of Pediatrics, Faculty of Medicine & Dentistry and School of Public Health, University of Alberta, Edmonton General Hospital, 8B19-11111 Jasper Avenue, Edmonton, AB, Canada T5K 0L4

## Abstract

*Background*. Use of complementary and alternative medicine (CAM) by children with cancer is high; however, pediatric best cases are rare. *Objectives*. To investigate whether best cases exist in pediatric oncology using a three-phase approach and to compare our methods with other such programs. *Methods*. In phase I, Children's Oncology Group (COG) oncologists were approached via email and asked to recall patients who were (i) under 18 when diagnosed with cancer, (ii) diagnosed between 1990 and 2006, (iii) had unexpectedly positive clinical outcome, and (iv) reported using CAM during or after cancer treatment. Phase II involved partnering with CAM research networks; patients who were self-identified as best cases were asked to submit reports completed in conjunction with their oncologists. Phase III extended this partnership to 200 CAM associations and training organizations. *Results*. In phase I, ten cases from three COG sites were submitted, and most involved use of traditional Chinese medicine to improve quality of life. Phases II and III did not yield further cases. *Conclusion*. Identification of best cases has been suggested as an important step in guiding CAM research. The CARE Best Case Series Program had limited success in identifying pediatric cases despite the three approaches we used.

## 1. Introduction

Complementary and alternative medicine (CAM) is popular in both adults and children [1]. Within the pediatric oncology population, CAM use has been reported as high as 84% [2–5], most commonly used as an adjunct to conventional medical treatment [6]. Pediatric CAM use has been associated with poor prognosis, as well as parental factors, such as their CAM use, age, and education [2]. Reasons reported for use are varied, such as to explore all possible treatment options [6]; enhance the efficacy or minimize side effects of conventional therapy [7]; boost immunity [6]; cure the cancer or slow its progression [6, 7]; and increase feelings of control over the child's treatment [7]. Many patients describe CAM as being helpful, and few report adverse effects [6, 8].

Despite the popularity of CAM, only half of parents disclose their child's CAM use to their physicians [3]. Less than half of pediatric oncologists inquire about CAM use, due to lack of time and knowledge or discomfort due to concern over harmful side effects [9]. The increased use of CAM in this vulnerable population reinforces the need for research into safety and efficacy to allow for informed decision making by physicians, patients, and families.

Several countries have developed initiatives to identify and investigate “exceptional cases” or “best cases” in cancer patients who use CAM [10]. One such program is hosted by the United States National Cancer Institute (NCI). In 1991, the NCI initiated the “Best Case Series Program” to evaluate potentially effective CAM therapies, with the goal of identifying those that warranted further research. In this program, cases are submitted by CAM practitioners to NCI who then conducts rigorous reviews of the case medical records. NCI Best Case criteria are among the most rigorous of the existing programs and include verification of a definitive diagnosis of cancer, documentation of disease response, and absence of confounders including concurrent conventional treatments and well-documented treatment history [11]. To our knowledge, the NCI Best Case Series Program inspired most similar existing programs. The NCI approach is recognized for yielding data with few confounders, while an acknowledged limitation is that exceptional results are framed only in terms of tumor regression, which does not take into consideration why oncology patients use CAM therapies.

Since NCI reported no pediatric best cases and since many children with cancer use CAM, we undertook a multistep approach to see if pediatric best cases could be identified. While our Pediatric Best Case Series was based on the NCI Best Case Series Program, we deliberately relaxed some of the criteria in order to be applicable to a pediatric population. Specifically, we allowed for concurrent use of CAM with conventional medical care, as it seemed unlikely that children would be permitted to forego conventional cancer treatment in favour of alternative therapies alone. Furthermore, we expanded the definition of a “best case” to include outcomes of interest to patients and families, such as prolonged survival and a markedly improved quality of life, as defined by respondents. Finally, in contrast to the NCI approach for case identification, we explored three different approaches to identify pediatric best cases in three steps: (i) through oncologists; (ii) through CAM research networks; and (iii) through CAM associations and colleges. 

## 2. Objectives

Our Pediatric Best Case Series had two main objectives: (i) to identify if “best cases” associated with CAM use existed in pediatric oncology and (ii) to compare the utility of three different approaches to case identification (steps I, II, and III). 

## 3. Methods

In step I, pediatric oncologists affiliated with the Children's Oncology Group (COG), including the 17 Canadian sites (C17), were contacted via email between January and April of 2007. Oncologists were asked to recall patients who met the following criteria: under 18 when diagnosed with cancer; diagnosed between 1990 and 2006; had, in their opinion, unexpectedly positive clinical outcome (as defined by tumor regression, prolonged survival, and/or improved quality of life); and reported CAM use during or after cancer treatment. 

Step II of the project involved partnering with CAM research networks. In November 2007 and February 2008, seven Canadian and US CAM networks were asked to advertise this study on their websites and list serves. CAM providers who learned of the study through these advertisements were asked, in turn, to notify their cancer patients of this study. Patients who were self-identified as best cases were asked to submit reports completed in conjunction with their oncologists.

Step III extended this partnership to include CAM associations and training organizations in Canada and the USA, representing the following modalities: acupuncture/acupressure, aromatherapy, art therapy, Ayurveda, chiropractic, herbalism, homeopathy, hypnotherapy, massage, music therapy, naturopathy, osteopathy, reflexology, reiki, therapeutic/healing touch, traditional Chinese medicine, and yoga. In September 2008 and February 2009, email notices were sent to over 100 Canadian and 100 US CAM associations and organizations asking them to advertise this study on their websites and list serves. CAM providers and patients were involved as for step II.

In order to identify similar programs around the world, a search strategy was developed using the snowball approach. Electronic databases, including Medline and Embase, were searched using the terms “Best Case Series” and “exceptional disease course”. References of identified articles were subsequently screened for further information. Coordinators or administrators of the identified programs were contacted through email for additional details regarding their programs. Submitted cases were compiled descriptively.

## 4. Results 

In step I, eleven cases from three COG sites were submitted. Two cases were excluded; one as it did not include a CAM therapy, and the other because the patient was 19 when diagnosed with cancer. The majority of the cases involved the use of traditional Chinese medicine (TCM) to improve quality of life (Table 1). Of note, seven cases were submitted by the CAM practitioner employed in the CAM treatment centre of the oncology hospital rather than the oncologist, as the oncologist referred the request directly to the CAM practitioner. These seven cases represented a small portion of the patients treated with CAM in their centre; however, due to time constraints, not all the cases were summarized and submitted. Steps II and III did not yield any additional cases.

## 5. Discussion 

Considering the widespread use of CAM by pediatric cancer patients, we anticipated that our Pediatric Best Case Series would reveal cases that had not been identified to NCI. In step I of this study, however, only two of more than 200 COG sites contributed valid cases. This approach had limited success in identifying pediatric best cases, as these cases were few in number and generally only related to improved quality of life. Since the majority of pediatric oncologists may not know about their patients' CAM use, we designed steps II and III to overcome this limitation, as the point of contact with patients then became CAM providers, not oncologists. Unfortunately, no further cases were identified, suggesting that either pediatric best cases do not exist or the approaches used in this study were unable to identify them. 

The limited response to our study may have been due to various methodological factors. In step I, we had limited response from pediatric oncologists. We believe their lack of response may have been multifactorial: (i) time constraints (they are potentially too busy to participate in a study such as this); (ii) lack of oncologist awareness of pediatric CAM use; and (iii) lack of willingness to believe that CAM might be beneficial for their patient population. While our approach was far less resource intensive than that used by NCI and involved oncologists to ensure data quality regarding verification of diagnosis and disease course, potential best cases may have been underestimated. A lack of communication between pediatric oncologists and their patients about patient CAM use exists [7, 9, 12]. Even when such use is discussed, it is possible that pediatric oncologists did not consider their patient's unexpected positive treatment results could be due to CAM, preferring attribution to chance or use of conventional therapies. Some pediatric oncologists responded to our inquiry in a very negative fashion, stating that they did not think CAM use has any potential for beneficial effect. Should this attitude be conveyed to their patients, it would likely hinder meaningful dialogue as patients would not disclose if they feel their decision to use CAM therapies will be questioned.

To overcome the issues faced in steps I, II, and III of our study, we focused on CAM providers to initiate the reporting process. However, in order to maintain data quality regarding verification of diagnosis and disease course, we asked that the pediatric oncologist participates in completing the case report form. Potential limitations of this approach include the apathy voiced by some CAM providers regarding the relevance of research to their practice. Data from a survey of CAM practitioners' conducted in Canada demonstrated that only 66% of chiropractors, 72% of naturopaths, and 51% of osteopaths felt that their practice benefited from advances in pediatric research [13]. Research is an onerous process, and it is unclear how many CAM providers are research users or feel comfortable with evidence-informed practice. Moreover, CAM providers may have wished to avoid scrutiny by pediatric oncologists or our research team, anticipating that their therapies may be assessed in a potentially prejudicial manner that discounted their effectiveness. Some CAM providers may have also simply wished to protect proprietary professional practices and therefore refrained from reporting best cases. Due to these limitations, we cannot determine if pediatric best cases in association with CAM use are elusive versus nonexistent. 

Several programs to identify best or exceptional cases in oncology have been initiated. A brief summary is found in Table 2. The greatest differences between programs consist of how cases are obtained and what criteria are used to define a “best case”. Based on our experience and the findings from other initiatives, we suggest a hybrid model that offers the combined strengths of the different programs: (i) the definition of “best cases” to be expanded based on outcomes chosen by patients, not only researchers or oncologists; this approach would support the inclusion of subjective and objective assessments of therapeutic effect (e.g., survival and quality of life, not only tumour regression); (ii) patients who use CAM concurrently with conventional medical treatment be eligible for study, as this is more reflective of real world practice and is therefore valuable, albeit confounded data. 

Our approach would be hypothesis generating, not hypothesis proving, and similar remarkable outcomes should be spontaneously reported in association with the same intervention; this could justify further study; (iii) collaboration between programs would promote communication and ideally result in standardization of criteria and methods as well as enhanced results; (iv) evaluating the potential to contact patients directly through patient centered organizations such as Canadian and US Cancer Societies; and (v) inclusion of the oncologist in the reporting process ensures data integrity with regards to diagnosis and expected course/prognosis and is far less exhaustive and resource intensive than the approach adopted by NCI. We would encourage the use of a mixed methods approach that includes qualitative interviews with CAM practitioners and oncologists in order to better understand the research field and to develop better hypotheses and methods to identify best cases in the future. 

Due to heterogeneity of reported results as well as missing results in the original reports, we are unable to compare outcomes of the various programs in terms of cases identified and classified as best cases. It is hoped that standardization of methods will allow for data comparison and combination in future best case reports. 

## 6. Conclusion

CAM use is extremely common amongst oncology patients, including children and youth. Despite multiple initiatives that try to identify the positive effects of CAM use, very few best cases have been identified thus far. Our study has evaluated the strengths and limitations of three different approaches to identify pediatric best cases. Since our greatest yield came from pediatric integrative oncology centers, where CAM is included in patient treatment plans, we have initiated discussions with North American pediatric integrative oncology centers, as identified through the Society for Integrative Oncology (SIO) and COG CAM Special Interest Group, to set up a collaborative Pediatric Best Case Series. Our hope is to identify these elusive cases and thereby generate hypotheses to support research that may benefit children with cancer.

## Figures and Tables

**Table 1:** Pediatric Best Case Series: step I.

Age at diagnosis; gender	Diagnosis	CAM treatment details	Overall outcome
12 years; F	High grade osteosarcoma of right distal femur	TCM acupuncture and Vaccaria seed patch treatments for pain and narcotic wean	Improved quality of life

3 years; M	Burkitt's lymphoma	TCM acupuncture, acupressure, Vaccaria seed patches, and herbal therapy (out-patient) for pain, anxiety, poor circulation, and feeding issues	Improved quality of life

14 months; F	Acute lymphoblast leukemia	TCM acupressure, massage, and Vaccaria seed patches for improving pain, appetite, insomnia	Improved quality of life

5 years; M	Neuroblastoma	TCM acupuncture, Vaccaria seed patches, moxibustion, and herbal therapy for pain, anxiety, appetite, fatigue, insomnia, and neurological deficits	Improved quality of life

4 years; M	Neurofibromatosis type 2	TCM acupuncture, acupressure, Vaccaria seed patches, and herbal therapy for severe, incapacitating headache, nausea, vomiting, and pain	Improved quality of life

11 years; M	Acute lymphoblast leukemia with lymphoma	TCM acupuncture, acupressure, Vaccaria patches, and massage to assist successful wean off ventilator, sedation, assist in rehabilitation, anxiety, fatigue, and weakness, poor appetite	Improved quality of life

15 years; F	Acute myeloid leukemia	TCM acupuncture, acupressure, seed patch, and massage for pain, anxiety, neuropathic pain, shortness of breath, tolerance, nausea, vomiting, poor appetite, insomnia, and depression	Improved quality of life

15 years; M	Metastatic fibrolamellar hepatocellular carcinoma	Multiple nonconventional treatments including coffee enemas and enzyme therapies	Temporary remission (6 months)

NR; NR	Brain tumor	Larch arabinogalactan for tolerance to narcotics	Did not have the expected cytopenias

**Table 2:** Best case programs—oncology cases.

Program/patient location/-scope/age/time frame	Primary goal	How best cases are submitted/obtained	How best cases are evaluated	Criteria of a best case	Program strengths
Studies					
Care, Edmonton; North America; pediatric; 2007/2008	To identify and prioritize CAM therapies that deserve further evaluation and promote partnerships that can successfully enable this goal.	Step I Active: email to directors of all North American COG sites (i) by oncologists. Step II Active: advertised via CAM research networks (ii) by CAM providers. Step III Active: advertised via CAM networks, associations, and training institutes (iii) by CAM providers	Step I Qualitative, descriptive summaries. Step II Study coordinators, review the number and nature of reports, and compare to phase I. Step III Study coordinators, review number and nature of reports, and compare to steps I and II.	Best Case to include: tumor regression, prolonged survival, and markedly improved quality of life.	Active solicitation; broad definition of best case; inclusion of multiple reporting/ID systems.

NCCAM, NIH, Bethesda; 2 clinics-Freeport, Bahamas (immunoaugmentation therapy (IAT), and New York, NY (naltrexone); age NR; 2001 [14]	Study used to determine if sufficient evidence is present to recommend further study.	Active: visit to sites (i) by CAM providers.	Patient records were obtained after receiving consents; patients were interviewed by telephone and screened based on the set criteria.	As for NCI (i) Documentation of diagnosis. (ii) Evaluation of proper antitumor endpoint. (iii) Absence of concurrent treatments.	Active solicitation.

Ulrik Dige, Denmark; Denmark; ages NR; dates NR [15]	Study conducted to explore exceptional cancer patients for further knowledge about CAM.	Active: media coverage (i) by patients.	As analysis of the cases;Dige conducted qualitative interviews and a thorough evaluation of medical hospital records.	Well documented improvement or total remission without conventional treatment.	Active solicitation.

Johanna Hök: Stockholm, Sweden; Sweden; all ages; 2004-2005 [16]	Aim of the thesis to explore perspectives on CAM use among individuals with cancer in connection to reported exceptional sickness trajectories.	Active: media coverage (i) by patients.	Patient interviews conducted along with evaluation of CAM reports using manifest content analysis and principal component analysis.	Cases were framed exceptional by the individuals reporting the case.	Active solicitation; broad definition of best case.

Programs					
National Cancer Institute (NCI), Bethesda; United States; all ages; 1991–current [10, 11]	To identify CAM approaches for cancer which warrant NCI-initiated prospective research.	Passive: (i) by CAM provider.	Relevant medical records documents, pathologic slides, and medical imaging studies reviewed by a Best-Case Series Review Team (1-2 physicians and 1-2 oncology nurses).	(i) Definitive diagnosis. (ii) Documented disease response. (iii) Absence of confounders. (iv) Documented treatment history.	Expert review of cases.

NAFKAM, Norway; Scandinavia; all ages; up to 2002–08/2010 [10, 17]	To develop a registry to facilitate research on patients who have exceptional disease courses/best and worse cases.	Passive: (i) by patient or by CAM provider.	Reviewed by NAFKAM's medical doctor to classify and assess case history.	(i) Experience of unusual treatment results after the use of CAM. (ii) Confirmed diagnosis before starting CAM treatments.	Broad definition of best case.

UCMO, Germany; Klinikum Nuernberg; ages NR; 1989–current [10, 18]	To decide whether purported tumor-specific efficacy of a CAM warrants further investigation.	Active: contact of CAM provided by UCMO.	Reviewed by coordinating M.D. and internal team of oncologists, radiologists, and pathologists.	As for NCI.	Active solicitation.

Hufeland Klinik Study, Germany; Hufeland Klinik, Bad Mergentheim, Germany; all ages; 1998/1999 [19]	Patients evaluated to help identify treatments that warranted further study.	Active: (i) by head practitioner selected cases for review.	Reviewers eliminated cases that did not fulfill best case criteria based on their summaries.	(i) Well documented diagnosis. (ii) Evidence of cancer when starting CAM. (iii) Unconventional treatment received according to CAM practitioner's regime. (iv) Absence of concurrent therapies.	Active solicitation.
